# The Binding of 3‐O‐Methylfluorescein Phosphate to the Catalytic Domain of the Human CDC25B Phosphatase: A Structural Investigation

**DOI:** 10.1002/cbic.202600010

**Published:** 2026-03-29

**Authors:** Romualdo Troisi, Rosario Rullo, Valeria Napolitano, Grzegorz M. Popowicz, Emmanuele De Vendittis, Filomena Sica

**Affiliations:** ^1^ Department of Chemical Sciences University of Naples Federico II Complesso Universitario di Monte Sant’Angelo Naples Italy; ^2^ Institute for the Animal Production Systems in the Mediterranean Environment Consiglio Nazionale delle Ricerche Portici Italy; ^3^ Institute of Structural Biology Helmholtz Zentrum München Neuherberg Germany; ^4^ Biomolecular NMR and Center for Integrated Protein Science Munich at Department Chemie Technical University of Munich Garching Germany; ^5^ Department of Molecular Medicine and Medical Biotechnologies University of Naples Federico II Naples Italy

**Keywords:** 3‐OMFP, CDC25B, crystal structure, ligand binding, structural basis for inhibitor design

## Abstract

The molecular mechanisms by which the human CDC25B activates the CDK1/cyclin B complex in the cell cycle, as well as how it can be inhibited by synthetic inhibitors at the atomic level, are still under investigation. Valuable insights have been gained from the molecular structure here‐described, which captures for the first time the interaction between the C‐terminal domain of the inactive mutant CDC25B C473S (CDC25B‐S) and the commonly used synthetic substrate 3‐O‐methylfluorescein phosphate (3‐OMFP). Crystallographic studies reveal that 3‐OMFP engages multiple residues within the active site and the adjacent “swimming pool” of CDC25B‐S, establishing specific interactions and prompting local adjustments in this region. These structural features explain the increased resistance to thermal denaturation of CDC25B‐S observed through circular dichroism measurements upon substrate binding. The structural changes induced by 3‐OMFP lead to a conformation comparable to that of CDC25A bound to its substrate, the CDK2/cyclin A complex. These findings qualify 3‐OMFP as a promising starting model for the rational design of selective competitive inhibitors of CDC25B having reduced off‐target effects.

## Graphical abstract

Structural studies on the complex that CDC25B C473S (CDC25B‐S) forms with the 3‐O‐methylfluorescein phosphate (3‐OMFP) reveal how the fluorogenic substrate binds CDC25B‐S, engaging key binding regions and inducing structural adjustments that stabilize the protein. These changes result in a protein conformation similar to that of CDC25A bound to the CDK2/cyclin A substrate and offer a structural basis for designing novel CDC25B inhibitors.

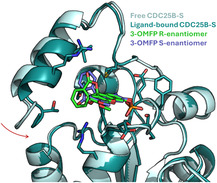

## 1 Introduction

Regulation of the cell cycle progression is a crucial process of living cells, which requires a fine control of its sequential steps, because their deregulation may lead to the insurgence of various cancer malignancies [1]. Among the macromolecules involved in this process, crucial roles are played by the cyclin‐dependent kinase (CDK) class of serine/threonine kinases complexed to specific proteins called cyclins [2, 3, 4, 5], and by the cell division cycle 25 (CDC25) dual phosphatases [6, 7]. Specifically, the activity of CDK/cyclin complexes is turned off by the process of phosphorylation of specific tyrosine and threonine residues within the CDK subunit [2]. On the other hand, dephosphorylation of the tyrosine and threonine residues in CDKs catalyzed by CDC25 dual phosphatases activates the CDK/cyclin complexes and the progression of cell cycle [8, 9, 10]. The pivotal role played by CDC25 in cell cycle is confirmed by the finding that these proteins are often overexpressed in human cancers [6, 11]. As a consequence, inhibition of these phosphatases represents a promising therapeutic approach in oncology, using CDC25 as an indirect target to turn off the CDK/cyclin complexes, thus inhibiting the cell cycle and the proliferation of damaged cells [1]. Moreover, since a long block of the cell cycle eventually leads the cell to apoptosis, CDC25 proteins, in addition to an anti‐proliferative effect, also possess a pro‐apoptotic effect [12]. All these findings confirm the usage of CDC25 phosphatases as promising targets in the treatment and diagnosis of cancer in the context of precision medicine, which aims at individualized care based on patients’ genetic and molecular profiles [13].

In mammalian cells, three CDC25 homologs have been identified and named CDC25A, CDC25B, and CDC25C [7, 14]. CDC25A is found to act at the G1/S transition, whereas CDC25B and CDC25C play important roles at the G2/M transition [8, 15] and have additional roles, including DNA damage repair and regulation of meiosis, respectively [13]. In particular, CDC25B promotes the G2/M phase transition by removing two inhibitory phosphate groups from the CDK1 kinase in the CDK1/cyclin B complex [8, 9, 10, 16].

From a structural point of view, all CDC25 proteins consist of two domains (N‐ and C‐terminal). The N‐terminal regulatory domain, poorly structured and highly variable in the three homologs, contains phosphorylation sites through which regulation occurs [17]. The mobility of the N‐terminal domain determines the ability of the protein to associate with different partners and adapt to the dynamic changes of the cell [18, 19]. The C‐terminal catalytic domain of CDC25 is highly conserved within the human CDC25 family and across eukaryotes. It is characterized by a small α/β domain with a central parallel β‐sheet surrounded by α‐helices [20, 21]. The active site region features the characteristic HCX_5_R motif, where the catalytic cysteine is located. X_5_ represents a ring composed of 5 residues (Glu‐Phe‐Ser–Ser‐Glu) whose amide hydrogens interact with the phosphate of the substrate [20, 21, 22]. The X‐ray crystal structures of the catalytic domain of CDC25A (PDB code: 1C25), CDC25B (PDB code: 1QB0), and CDC25C (PDB code: 3OP3) show significant differences in their C‐terminal regions (Figure S1). Comparison of the three structures shows that the C‐terminal region of CDC25B forms an α‐helix that bends toward the active site, thereby limiting its solvent exposure. This C‐terminal helical conformation is absent in CDC25A and CDC25C crystal structures. The recently reported cryogenic electron microscopy (cryo‐EM) structure of the full‐length catalytic domain of CDC25A in complex with CDK2‐cyclin A (PDB code: 8ROZ) has revealed that the C‐terminal region of CDC25A forms an α‐helix (Figure S1), which is longer than that present in the crystal structure of the C‐terminal catalytic domain of CDC25B and has a critical role in the interaction with the CDK2‐cyclin A substrate [23]. Long C‐terminal helices are also present in the predicted models of the three full‐length isoforms from the AlphaFold Protein Structure Database (Figure S1).

Over the past years, several synthetic and natural CDC25B inhibitors with different structural features and belonging to various chemical classes, including phosphate bioisosteres, electrophilic entities, and quinonoids, have been reported [12, 19, 24, 25, 26, 27, 28, 29, 30, 31, 32, 33]. However, only two crystal structures of complexes between CDC25B and ligands have been deposited in the Protein Data Bank (PDB) to date (PDB codes: 4WH7, 4WH9). In these structures, the two ligands bind to an enzyme pocket distant from the active site and adjacent to the protein–protein interaction interface with CDK2/cyclin A substrate [34].

As a useful starting point for the design of new powerful competitive inhibitors of CDC25B, we determined the X‐ray crystal structure of the complex formed between the catalytically inactive substrate‐trapping mutant, CDC25B‐S, obtained by replacing the active‐site Cys473 with a serine, and the fluorogenic substrate 3‐O‐methylfluorescein phosphate (3‐OMFP), which displays moderate affinity for CDC25B‐S (*K*’_D_ = 1.23 µM) [35]. In addition, we solved the crystal structure of the free CDC25B‐S at a higher resolution (1.34 Å) than those previously available in the PDB. Circular dichroism (CD) measurements have been implemented to evaluate the in–solution interaction between CDC25B‐S and 3‐OMFP substrate.

## 2 Results and Discussion

### 2.1 Spectroscopic Analysis

The effect of the C473S replacement in CDC25B and of the interaction with the fluorogenic compound 3‐OMFP, widely used as a synthetic substrate of all CDC25 isoforms [35, 36, 37, 38, 39], on the secondary structure of the protein has been evaluated by CD analysis.

**FIGURE 1:**
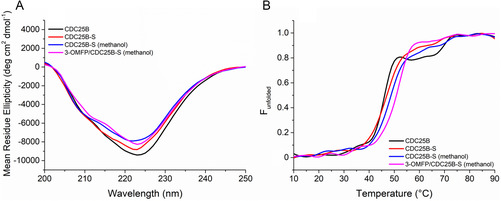
(A) CD spectra and (B) thermal denaturation curves of CDC25B (black line), CDC25B‐S (red line), CDC25B‐S in the presence of 2.3% v/v CH_3_OH (blue line), and CDC25B‐S in the presence of a fivefold molar excess of 3‐OMFP (magenta line). Solutions were prepared in 40 mM Tris‐HCl pH 8.0, 40 mM NaCl, and 1.7 mM dithiothreitol (DTT) at a protein concentration of 0.2 mg mL^−1^. The 3‐OMFP‐containing sample included 2.3% v/v CH_3_OH, derived from the ligand solution. Spectra were recorded at 10°C. Denaturation curves are shown as the fraction of unfolded protein, obtained by monitoring the signal intensity at 222 nm, as a function of temperature.

Using experimental conditions reproducing those of the biological activity assays [35], CD spectra were recorded on the purified recombinant C‐terminal domain of CDC25B and CDC25B‐S. The comparative spectroscopic analysis suggested that the C473S mutation does not cause significant changes in the secondary structure of CDC25B (Figure 1A). CD spectral features are characteristic of a protein having a mixed α‐helix and β‐sheet secondary structure, with a minimum at 222 nm and a shoulder at 210 nm. To verify the effect of the Cys473 replacement on the protein thermal unfolding behavior, the signal intensity at 222 nm was followed at increasing temperature from 10°C to 90°C. The resulting melting temperatures (*T*_m_) for CDC25B and CDC25B‐S were 46°C and 47°C, respectively, thus indicating that the two proteins exhibit a comparable resistance to thermal denaturation (Figure 1B and Table S1). Under these experimental conditions, the denaturation process is irreversible for both proteins, as assessed by the lack of signal recovery upon cooling (data not shown). Moreover, the appearance of sample opalescence suggests unfolding coupled to aggregation in both CDC25B and CDC25B‐S. Then, the thermal unfolding of CDC25B‐S was evaluated upon the addition of 3‐OMFP whose solubilization required the presence of 2.3% v/v CH_3_OH in the experimental conditions. However, this alcohol only induced minimal changes in the secondary structure (Figure 1A) and the *T*_m_ (48°C, Figure 1B and Table S1) of CDC25B‐S. In the presence of fivefold molar excess of substrate (Figure 1A), the secondary structure of CDC25B‐S was substantially unaltered, whereas the denaturation temperature increased to 52°C (Figure 1B and Table S1), indicating the effective interaction of the two molecules leading to an increased resistance to the thermal unfolding of the protein, with a ligand‐dependent delay of the irreversible denaturation process.

### 2.2 Crystallographic Analysis

Crystals of the free CDC25B‐S were obtained essentially under previously reported conditions [21]. They diffracted X‐rays to a resolution of 1.34 Å (see Table S2 for data collection and refinement statistics), which is higher than that of other CDC25B structures available in the PDB.

**FIGURE 2:**
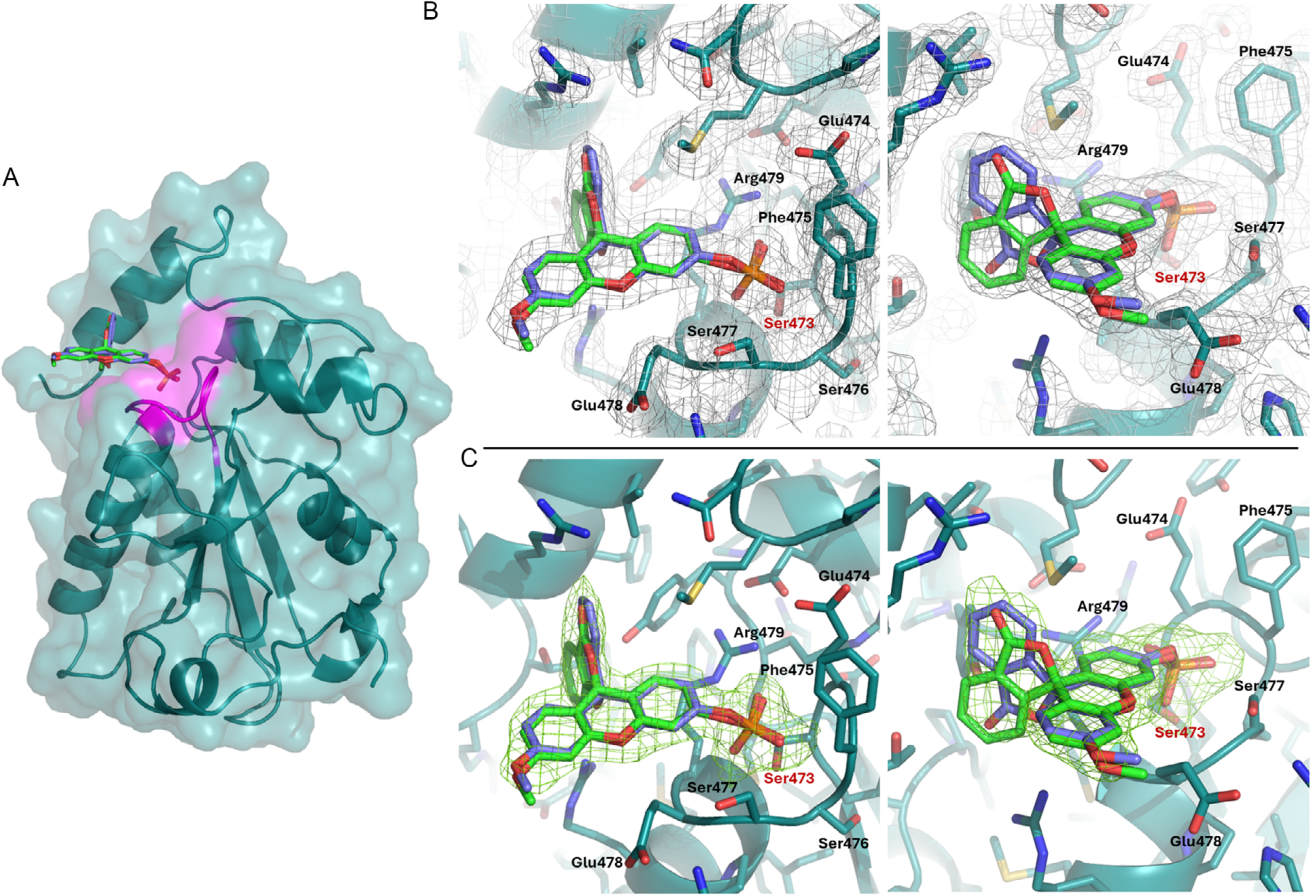
(A) Surface/cartoon/stick representation of the crystal structure of the 3‐OMFP/CDC25B‐S complex. The HSX_5_R motif of the active site is in magenta. (B) 2F_o_‐F_c_ electron density map (gray) of the 3‐OMFP molecule bound to the active site of CDC25B‐S protein contoured at 1.0 σ level. (C) Omit F_o_‐F_c_ electron density map (green) of 3‐OMFP contoured at 3.0 σ level. Both maps are shown in two orientations related by *a* ∼90° rotation. The Ser473 residue, which replaces the catalytic cysteine, is labeled in red. In all panels, the R‐ and S‐enantiomers of 3‐OMFP are green and violet, respectively.

After an extensive search of the crystallization conditions, co‐crystals of the 3‐OMFP/CDC25B‐S complex were obtained under conditions never reported before for CDC25 proteins. In particular, crystals (Figure S2) appeared within a few days at 20°C in a solution containing 3.8 M NaCl, 0.1 M HEPES pH 7.5, and 1.2% v/v 2‐propanol using the hanging‐drop vapor diffusion method. X‐ray diffraction data were collected on the cryoprotected crystals at 100 K on the X06 DA‐PXIII beamline of the Swiss Light Source (SLS) (see Table S2 for data collection statistics). Crystals belong to the space group P2_1_2_1_2_1_, diffract X‐rays at 2.04 Å resolution, and present a single CDC25B‐S polypeptide chain in the asymmetric unit. The structure was solved by the molecular replacement method using the Phaser MR program [40] and the coordinates of CDC25B‐S from the PDB code 2A2K [41], stripped of all its ligands, as the search model. The final model (Figure 2A), partially automatically built using ARP/wARP program [42], was refined using the REFMAC5 program [43] to *R*_factor_ = 0.195 and *R*_free_ = 0.214 with good stereochemistry (see Table S2 for refinement statistics). Deviations from ideal bond lengths and angles are 0.004 Å and 1.430°, respectively. Notably, the overall conformation of the protein is not significantly affected by 3‐OMFP binding (Figure S3): the Cα root–mean‐square deviation for residues 377–549 of the 3‐OMFP/CDC25B‐S complex from the starting model (PDB code: 2A2K) and from the here newly reported crystal structure of the free CDC25B‐S is 0.62 Å and 0.78 Å, respectively. This observation is consistent with the CD data (Figure 1A), which suggest that ligand binding does not lead to appreciable alterations in the global secondary structure of the protein. When comparing the crystal structures of the free and the 3‐OMFP‐bound CDC25B‐S, the most significant variations are confined to the flexible N‐terminal expression tag, which precedes the actual protein sequence starting at Glu377, from which no major structural differences have been detected (Figure S3). These variations are likely due to differences in crystal packing and reflect the intrinsic flexibility of this region. Notably, in the structure of the 3‐OMFP/CDC25B‐S complex, an additional portion of the expression tag (Ser364‐Arg370) could be modeled, whereas this region was not observed in the structure of the ligand‐free protein.

Inspection of the difference Fourier (2F_o_‐F_c_ and F_o_‐F_c_) electron density maps clearly revealed the presence of the 3‐OMFP molecule within the active site region (Figure 2B), in proximity to the main chain of residues 474–478 and the side chains of Arg479 and Ser473, which in CDC25B‐S replaces the functional cysteine of the wild‐type protein. A comparison between the 2F_o_‐F_c_ electron density maps of the active site in the structure of the 3‐OMFP/CDC25B‐S complex and in the free CDC25B‐S is reported in Figure S4.

3‐OMFP is a fluorescein derivative in which the phenolic hydroxyl groups are chemically protected (methylated on one side and phosphorylated on the other). Based on this chemical structure, and in line with evidence that acylation or alkylation of phenolic groups can lock fluorescein derivatives into a lactone state [44], 3‐OMFP is expected to be more stable in the “closed” lactone form (Figure S5A) than the “open” cationic form (Figure S5B). Interestingly, 3‐OMFP exhibits minimal or no fluorescence [45, 46], whereas a strong emission is observed after enzymatic phosphate cleavage, when the 3‐O‐methylfluorescein (3‐OMF) adopts a highly rigid and conjugated “open” quinoid structure (Figure S5C). This property underlies the fluorogenic assays that employ 3‐OMFP substrate to monitor the activity of this protein family [47]. Although the resolution of the 3‐OMFP/CDC25B‐S structure (2.04 Å) does not allow an unambiguous assignment of the chemical form adopted by the substrate, the planar and continuous electron density observed for the lateral aromatic‐COO substituent seems more consistent with the expected cyclic lactone form than with the unstable cationic form (Figure S6). Considering the chirality of the lactone form of 3‐OMFP (Figure S7), both enantiomers of the ligand were modeled at the binding site with equal occupancies of 0.5. In fact, the omit F_o_‐F_c_ electron density map obtained by removing 3‐OMFP does not allow discrimination between the two enantiomers (Figure 2C). Both models fit well within the active site, and no steric clashes were detected. Mean B values of both enantiomers (R‐enantiomer: 36.5 Å^2^; S‐enantiomer: 33.1 Å^2^) are in line with that of the whole structure (36.9 Å^2^, Table S2). This interpretation is consistent with the broad solvent‐exposed binding site of the protein and with the dominant role of the phosphate group of 3‐OMFP in ligand binding. Moreover, the presence of both enantiomers at the binding site gives additional reliability to previously reported kinetic studies [36], which would otherwise be inaccurate if only one enantiomer was selectively bound.

**FIGURE 3:**
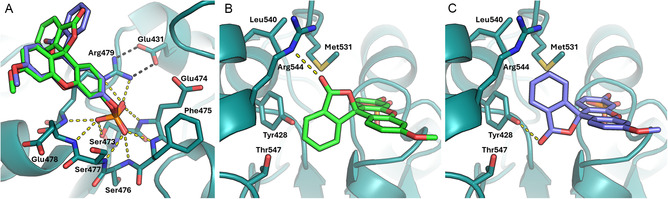
Contacts between CDC25B‐S (deep teal) and 3‐OMFP (R‐enantiomer in green and S‐enantiomer in violet). (A) Hydrogen bonds (yellow dashed lines) connecting the 3‐OMFP phosphate group and CDC25B‐S residues in the range 473–479. The correct orientation of the Arg479 sidechain for interaction with 3‐OMFP is stabilized by an intramolecular salt bridge with Glu431 (black dashed lines). (B,C) Bicyclic lactone group of 3‐OMFP (R‐enantiomer in panel B; S‐enantiomer in panel C in the open cleft formed by Tyr428, Met531, Leu540, Arg544, and Thr547. Interactions of both enantiomers with some of these residues are shown (yellow dashed lines).

The interface area of both enantiomers of 3‐OMFP with CDC25B‐S is around 288 Å^2^ (Table S3) and the primary binding of the ligand phosphate group engages the backbone nitrogens and/or the side chains of the SX_5_R (Ser473‐Glu474‐Phe475‐Ser476‐Ser477‐Glu478‐Arg479) ring motif (Figure 3A and Table S3). In detail, one non‐bridging oxygen of this phosphate group is positioned within hydrogen‐bonding distance from the Nε and one Nη of the Arg479, while another interacts with the hydroxyl group of Ser473. The average distances are 2.7 Å for the R‐enantiomer and 2.9 Å for the S‐enantiomer. Moreover, these two oxygen atoms of 3‐OMFP molecule are on average at 2.9 Å from the backbone nitrogen atoms of Glu474, Glu478, and Arg479. Specifically, one oxygen atom is held between the side chain of Ser473 and the backbone nitrogens of Glu478 and Arg479, and the other between the side chain of Arg479 and the backbone nitrogen of Glu474 (Figure 3A). The third non‐bridging oxygen is at a similar average distance from the three backbone nitrogen atoms of Phe475, Ser476, and Ser477 (Figure 3A). It is interesting to note that the phosphate group of 3‐OMFP does not fully overlap with the sulfate ion observed in the crystal structure of the ligand‐free CDC25B‐S (Figure S8A). The sulfate, originating from crystallization conditions, maximizes its contacts with the side chain of Arg479 by coordinating two of its oxygens, while another oxygen is shared between the hydroxyl group of Ser473 and the backbone nitrogens of Ser476, Ser477, and Glu478 (Figure S9A). The interaction network also slightly differs from that observed for the CDK2 phosphorylated tyrosine in the ternary complex [23] that CDC25A forms with the CDK2‐cyclin A substrate (Figures S8B and S9B). These findings reflect the different nature of the ligands.

**FIGURE 4:**
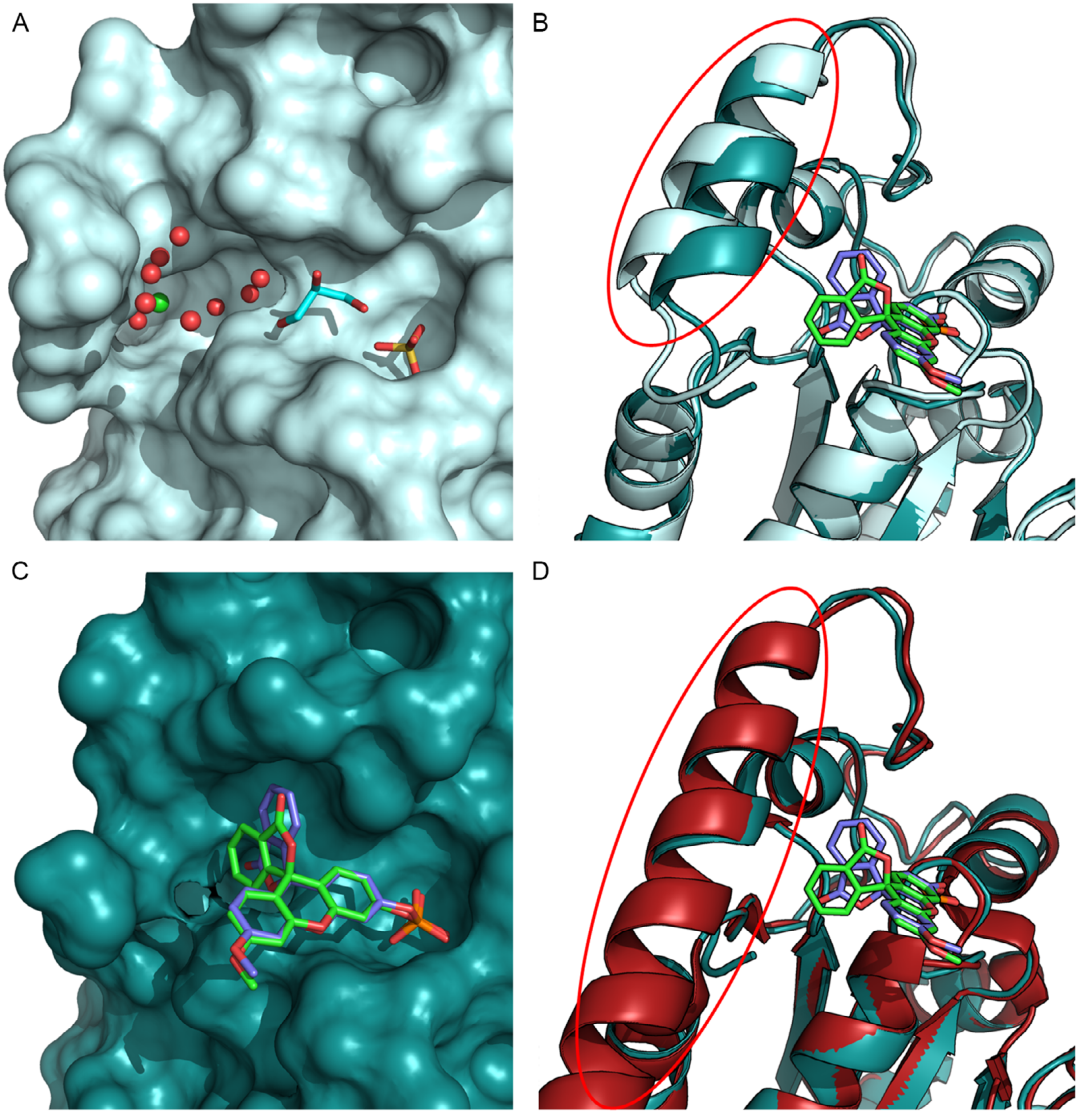
(A,C) Surface representation of the active site (right) and “swimming pool” cavity (left) of CDC25B‐S in (A) ligand‐free form (light cyan) and (C) 3‐OMFP‐bound state (deep teal). In (A), water molecules (red spheres) and a chloride ion (green sphere) are visible in the “swimming pool”, while the active site contains a bound sulfate ion and a nearby glycerol molecule from the cryo‐protection procedure (both shown as sticks). (B,D) Zoom‐in view of the superimposed cartoon representations of the 3‐OMFP/CDC25B‐S complex (deep teal) with B) ligand‐free CDC25B‐S (light cyan) and (D) CDC25A (dark red) when bound to CDK2–cyclin A (PDB code: 8ROZ). The C‐terminal helices are highlighted by circles. In (B–D), the R‐ and S‐enantiomers of 3‐OMFP are shown in green and violet, respectively.

The methylfluorescein moiety of 3‐OMFP moves away from the protein surface with its bicyclic lactone group laying against an open cleft formed by Tyr428, Met531, Leu540, Arg544, and Thr547 (Figure 3B, C). In the ligand‐free protein, some of these residues define a deep and large cavity adjacent to the catalytic pocket. This cavity is called “swimming pool” [48, 49] because of the abundance of well‐ordered water molecules it contains in the absence of the substrate (Figure 4A). Notably, Leu540, Arg544, and Thr547 are part of the CDC25B‐S C‐terminal helix (Lys537–Thr547), which undergoes a sort of rigid‐body motion toward the ligand when compared with the unbound structure (Figure 4B). This movement results in a narrowing and reshaping of the “swimming pool” cavity (Figure 4C). Arg544 exhibits the most pronounced conformational rearrangement of its side chain, enabling accommodation of the bicyclic lactone group on the cleft (Figure S10). In both 3‐OMFP enantiomers, the lactone carbonylic oxygen is within a hydrogen‐bonding distance from the side chain of one of the cleft residues (Table S3). Specifically, in the R‐enantiomer (Figure 3B), the carbonyl oxygen is 3.2 Å from the Nε of Arg544, whereas in the S‐enantiomer (Figure 3C), it is 2.9 Å from the hydroxyl group of Tyr428. In the latter enantiomer, the ring oxygen of the lactone group lies 3.0 Å from the Nη of Arg482 (Figure S11). This contact, however, should be considered with care, as the electron density observed for the Arg482 side chain is poorly defined, suggesting that the interaction may be transient. The contribution of hydrophobic interactions is overall modest (Table S3), involving the bicyclic lactone group within the cleft and additional contacts from the inner portion of the ligand facing Glu478 and Arg479. This reflects both the predominantly polar nature of the CDC25B‐S interacting residues and the high solvent exposure of the ligand (Figure S12). In fact, in both enantiomers, the side of the ligand opposite to Arg482 does not interact with the protein, but it engages a crystallographic symmetry mate through hydrophobic interactions (Figure S13). The resulting interface, covering approximately 160 Å^2^, contributes to crystal packing contacts that are distinct from those of the ligand‐free protein, while retaining the same space group (Table S2).

3‐OMFP can therefore be considered as a bivalent ligand that, predominantly through polar contacts, simultaneously targets the active site and the residues lining the “swimming pool”, which rearranges upon ligand binding. In particular, the lactone ring of the S‐enantiomer contacts Tyr428 and Arg482 similarly to NSC 663284, a potent, selective, and cell‐permeable quinolinedione CDC25 phosphatase inhibitor interacting with the “swimming pool” residues [33, 50, 51]. Interestingly, in the 3‐OMFP/CDC25B‐S structure, the backbone conformation of the C‐terminal helix more closely resembles that observed in CDC25A when bound to CDK2‐cyclin A module [23] (Figure 4D), than the conformation found in the free CDC25B‐S (Figure 4B). This indicates that a small ligand such as 3‐OMFP is able to induce in the C‐terminal helix a movement comparable to that produced by the recognition of the CDK‐cyclin substrate.

## 3 Conclusion

Summarizing, we have, for the first time, solved and refined the three‐dimensional structure of the complex formed by the catalytic domain of CDC25B‐S protein and the synthetic substrate 3‐OMFP. Furthermore, we have investigated the effect of this binding on the protein unfolding process through CD spectroscopy. The key findings of this study can be summed up as follows:

(i) The direct structural evidence of 3‐OMFP binding to the dual‐specificity phosphatase CDC25B‐S has been reported and compared with a newly solved high‐resolution structure of the unbound protein. The 3‐OMFP is observed in its favored “closed” lactone form, and both its enantiomers could be modeled, differing only in the region beyond the chiral center. While other deposited PDB structures report interactions of different proteins with fluorescein or its derivatives in different chemical states—cationic (PDB codes: 1DZH, 1MPA, 2MPA, 7AUY, 7AV5), quinoid (PDB codes: 1FLR, 1N0S, 1T66, 1X9Q, 2A9N, 2F14, 2NMV, 3M2J, 4BX7, 4FAB, 4ZS2, 4ZS3, 5DYO, 5IEL, 5TSV, 5TSX, 6AYL), or lactone (PDB codes: 2FDC, 2XN6, 2XN7, 7QEA)—to our knowledge this is the first structural characterization of 3‐OMFP bound to a phosphatase. The phosphate group is anchored within the active site by sidechain and backbone contacts involving all residues of the SX_5_R ring motif. In particular, Ser473 occupies the position of the catalytic cysteine in wild‐type CDC25B, resulting in a conformation that may closely resemble the substrate‐bound state prior to phosphate cleavage. The methylfluorescein moiety of 3‐OMFP locates its bicyclic lactone group on the open cleft formed by Tyr428, Met531, Leu540, Arg544, and Thr547 in proximity of the protein “swimming pool”. Contacts are established between the carbonyl oxygen of this moiety and some of these residues, while the rest of the methylfluorescein portion extends away from the protein surface.

(ii) Binding of 3‐OMFP to CDC25B‐S induces a slight rigid‐body motion of the C‐terminal helix backbone toward the protein surface, without causing major changes in the overall protein conformation. This subtle movement allows the accommodation of the bicyclic lactone group of 3‐OMFP, shaping the abovementioned cleft. It should be noted that the catalytic domain used in our studies corresponds to the Glu377‐Trp550 region of the full‐length CDC25B and therefore lacks the 16 C‐terminal residues, which in previous reports have been observed as part of a highly flexible tail [52]. Despite this cleavage, the C‐terminal helix of the complex under study adopts a conformation similar to that of the same region of CDC25A in the complex with the CDK2/cyclin A substrate [23]. The lacking terminal region of CDC25B could partially occlude the active site and form transient contacts with the protein core, potentially modulating substrate access and binding dynamics [52]. Hence, this tail could potentially engage additional interactions with natural substrates or designed inhibitors.

(iii) All these structural features may account for the increased resistance to thermal denaturation of the protein in the presence of excess 3‐OMFP, indicating that the here‐described crystal structure offers a valid representation of the interactions occurring in solution.

(iv) The ability of 3‐OMFP to simultaneously recognize the active site and the reshaped “swimming pool”, two regions hitherto used to obtain effective CDC25B inhibitors [29, 31, 33], makes this ligand an ideal template for the design of new inhibitors. In fact, despite the numerous inhibitors examined so far, it has not yet been possible to select a molecule that combines high affinity, specificity, reduced toxicity, and favorable ADME (absorption, distribution, metabolism, excretion) properties, such as bioavailability and cytotoxicity [53].

Collectively, this work provides solid evidence that a small phosphorylated molecule produces in the catalytic domain of a CDC25 enzyme structural effects quite similar to those of the effective substrate. Consequently, the here‐described structure not only enriches the still limited repertoire of ligand/CDC25B complexes but also offers a reliable framework for the rational design of CDC25B competitive inhibitors and supports the interpretation of solution studies aimed at understanding the protein interactions with substrates and/or inhibitors.

## 4 Experimental Section

### 4.1 Materials

3‐O‐Methylfluorescein phosphate (3‐OMFP), isopropyl‐β‐thiogalactopyranoside (IPTG), dithiothreitol (DTT), and tris(2‐carboxyethyl)phosphine hydrochloride (TCEP) were purchased from Merck (Sigma–Aldrich). A stock solution of 3‐OMFP was prepared in methanol at a concentration of 2 mM. All other chemicals and solvents were of analytical grade or higher.

### 4.2 Protein Preparation

Preparation of the recombinant C‐terminal domain of wild‐type CDC25B and its mutant form (CDC25B‐S), carrying the C473S replacement, was carried out as previously reported for wild‐type [31] and mutant [35] proteins. In particular, the expression system was constituted by the *Escherichia coli* BL21(DE3) strain from Novagen (Madison, WI, USA) and the vector pET28a‐CDC25B‐cd or pET28a‐CDC25B‐C473S‐cd, the latter containing the nucleotide mutation leading to the C473S amino acid replacement. In particular, the truncated constructs of both CDC25B and CDC25B‐S comprise residues Glu377 to Trp550, according to the numbering of the full‐length CDC25B, and include an N‐terminal expression tag containing a poly‐His sequence used for purification. The purified samples of CDC25B and CDC25B‐S, obtained through the Ni^2+^agarose affinity chromatography, were homogeneous when analyzed by SDS polyacrylamide gel electrophoresis or RP‐HPLC on a C4 column. As previously verified [35], CDC25B‐S was unable to hydrolyze the synthetic substrate 3‐OMFP.

### 4.3 Circular Dichroism (CD) Measurements

CD spectra were recorded with a Jasco J‐1500 spectropolarimeter equipped with a Peltier temperature controller. Far‐UV measurements were carried out at a protein concentration of 0.2 mg mL^−1^ in 40 mM Tris‐HCl pH 8.0, 40 mM NaCl, and 1.7 mM DTT at 10°C, using a cell with an optical path length of 0.1 cm. The substrate‐containing sample included 2.3% v/v CH_3_OH; the same concentration was also used in free protein experiments, where indicated. Spectra, registered with a 50 nm min^−1^ scanning speed, 2 s response time, 1 nm data pitch, and 2.0 nm bandwidth, were obtained by averaging three scans. Thermal unfolding curves were obtained by following the CD signal at 222 nm, in the 10°C–90°C range at a heating rate of 1.0°C min^−1^. The melting temperatures (*T*_m_) were determined through the analysis of the first derivative of the melting curve. The samples were cooled back to 10°C and the CD spectra were re‐scanned in order to evaluate the reversibility of the thermal denaturation.

### 4.4 Crystallization and Cryocooling

CDC25B‐S was concentrated up to 15 mg mL^−1^ in 10 mM Tris‐HCl pH 7.8, and 0.5 mM TCEP. Crystals of the free CDC25B‐S were grown at 4°C, as previously described [21], in 2.0 M ammonium sulfate, 0.5 M NaCl, 0.1 M Tris‐HCl pH 7.5, and 0.5 mM TCEP, using the hanging drop vapor diffusion method. 0.5 μL of protein solution was mixed with 0.5 μL of reservoir solution.

The complex formed by CDC25B‐S and 3‐OMFP was prepared by incubating at 4°C for 1 h the diluted protein (0.1 mg mL^−1^) with a 50‐fold molar excess of substrate in 40 mM Tris‐HCl pH 8.0, 40 mM NaCl, 10% v/v methanol, 3% v/v glycerol, and 1.7 mM DTT. Finally, the 3‐OMFP/CDC25B‐S complex was concentrated up to 9 mg mL^−1^.

An initial extensive screening of sitting‐drop crystallization experiments at 20°C and 4°C in 96‐well plates was carried out using a Mosquito Crystal protein crystallization robot (SPT Labtech) and commercially available crystallization screens (NeXtal QIAGEN Classics, Classics II, AmSO4, Anions, Cations, Nucleix, and JCSG+; Jena Bioscience JBScreen PACT++; Hampton Research PEGRx HT).

The optimization of the starting conditions was performed by hanging drop vapor diffusion method, mixing 0.5 μL complex solution with 0.5 μL reservoir solution. Crystals suitable for X‐ray diffraction data collection grew at 20°C in 3.8 M NaCl, 0.1 M HEPES pH 7.5, and 1.2% v/v 2‐propanol.

The crystals of the free CDC25B‐S and 3‐OMFP/CDC25B‐S complex were cryoprotected by adding 20% v/v glycerol and 25% v/v ethylene glycol to the crystallization solution, respectively, before being flash‐cooled in liquid N_2_.

### 4.5 Data Collection, Structure Determination, Refinement, and Structural Analysis

Diffraction data of free CDC25B‐S were collected at the ID30A‐1/MASSIF‐1 beamline of the European Synchrotron Radiation Facility (ESRF) at 1.34 Å resolution, using *λ* = 0.9660 Å. Those of 3‐OMFP/CDC25B‐S complex were collected at the X06DA‐PXIII beamline of the Swiss Light Source (SLS) at 2.04 Å resolution, using *λ* = 1.0000 Å. The datasets were processed using the autoPROC program [54, 55, 56, 57, 58]. Initial phases were determined by molecular replacement using Phaser MR [40] and the PDB entry 2A2K as a template. REFMAC5 [43] and Coot [59] programs were used for refinement and model building, respectively. In the case of 3‐OMFP/CDC25B‐S complex, partial modeling was automatically performed using ARP/wARP program [42]. The analysis of the Fourier difference (2F_o_‐F_c_, F_o_‐F_c_) electron density maps allowed to unambiguously place the 3‐OMFP ligand in the structure using Coot [59]. Both structures were validated using the Coot [59] routines and the PDB validation server (https://validate‐rcsb‐1.wwpdb.org/). Data collection and refinement statistics are reported in Table S2. The final coordinates and the structure factors were deposited in the Protein Data Bank (free CDC25B‐S = PDB code 9T09; 3‐OMFP/CDC25B‐S complex = PDB code 9T0A). The Superpose program from CCP4 package [56] was used to calculate the root‐mean‐square deviations (RMSD). The interface area of the 3‐OMFP/CDC25B‐S complex was examined using the PISA program [60] available online (https://www.ebi.ac.uk/pdbe/pisa/). All interactions within 3.5 Å between the two molecules were identified using the Contact program from the CCP4 package [56] and subsequently verified through visual inspection of the structure with Coot [59]. Hydrophobic interactions were identified using LIGPLOT (LigPlot^+^ package) [61]. Molecular graphics figures were rendered with PyMOL (DeLano Scientific, Palo Alto, CA, USA).

## Supporting Information

Additional supporting information can be found online in the Supporting Information section. **Supporting Table S1**: Melting temperature of CDC25B and its mutant CDC25B‐S in different conditions. **Supporting Table S2**: Data collection and refinement statistics. **Supporting Table S3**: Interface interactions and buried area in 3‐OMFP/CDC25B‐S complex. **Supporting Figure S1**: Experimental structures and predicted models of the catalytic domains of CDC25A, CDC25B, and CDC25C. **Supporting Figure S2**: Crystals of the 3‐OMFP/CDC25B‐S complex. **Supporting Figure S3**: Superimposition of the structures reported here for the 3‐OMFP/CDC25B‐S complex and the free CDC25B‐S protein. **Supporting Figure S4**: Details of the active site in the structures of the 3‐OMFP/CDC25B‐S complex and the ligand‐free CDC25B‐S protein. **Supporting Figure S5**: Chemical structures of 3‐OMFP in the “closed” lactone form and “open” cationic form, and chemical structure of the “open” quinoid form of the highly fluorescent 3‐OMF. **Supporting Figure S6.** Detail of the 3‐OMFP/CDC25B‐S structure in which the 3‐OMFP molecule has been modeled in an “open” cationic form. **Supporting Figure S7**: Chemical structures of the two enantiomers of 3‐OMFP lactone form. **Supporting Figure S8**: Comparison of the binding mode of the phosphate group of 3‐OMFP in 3‐OMFP/CDC25B‐S structure with the sulfate ion observed in the ligand‐free CDC25B‐S crystal structure and the phosphorylated tyrosine of CDK2 in the cryo‐EM structure of the CDK2‐cyclin A‐CDC25A complex. **Supporting Figure S9**: Contacts between the CDC25B‐S active site residues and the sulfate ion observed in the ligand‐free CDC25B‐S crystal structure, and the CDC25A active site residues and the CDK2 phosphorylated tyrosine in the cryo‐EM structure of the CDK2‐cyclin A‐CDC25A complex. **Supporting Figure S10**: Superimposition of the 3‐OMFP/CDC25B‐S complex and the free CDC25B‐S protein, highlighting the conformational rearrangement of Arg544 side chain to accommodate the bicyclic lactone group of 3‐OMFP. **Supporting Figure S11**: Transient interaction between the S‐enantiomer of 3‐OMFP and Arg482 of CDC25B‐S. **Supporting Figure S12**: The shallow surface active‐site pocket of CDC25B‐S: details of the binding of the R‐ and S‐enantiomers of 3‐OMFP, highlighting the high solvent exposure of the ligand and the predominantly polar nature of the interacting protein residues. **Supporting Figure S13**: Crystal packing contacts involving the 3‐OMFP molecule.

## Conflicts of Interest

The authors declare no conflicts of interest.

## Supporting information

Supplementary Material

## Data Availability

Atomic coordinates and structure factors for free CDC25B‐S and 3‐OMFP/CDC25B‐S complex have been deposited with the Protein Data Bank under accession numbers 9T09 and 9T0A, respectively.

## References

[1] S. Diaz‐Moralli , M. Tarrado‐Castellarnau , A. Miranda , and M. Cascante , “Targeting Cell Cycle Regulation in Cancer Therapy,” Pharmacology & Therapeutics 138 (2013): 255–271. 23356980 10.1016/j.pharmthera.2013.01.011

[2] D. O. Morgan , “Principles of CDK Regulation,” Nature 374 (1995): 131–134. 7877684 10.1038/374131a0

[3] M. Malumbres and M. Barbacid , “Mammalian Cyclin‐Dependent Kinases,” Trends in Biochemical Sciences 30 (2005): 630–641. 16236519 10.1016/j.tibs.2005.09.005

[4] L. Ding , J. Cao , W. Lin , et al., “The Roles of Cyclin‐Dependent Kinases in Cell‐Cycle Progression and Therapeutic Strategies in Human Breast Cancer,” International Journal of Molecular Sciences 21 (2020): 1960. 32183020 10.3390/ijms21061960 PMC7139603

[5] S. Ghafouri‐Fard , T. Khoshbakht , B. M. Hussen , et al., “A Review on the Role of Cyclin Dependent Kinases in Cancers,” Cancer Cell International 22 (2022): 325. 36266723 10.1186/s12935-022-02747-z PMC9583502

[6] K. Kristjánsdóttir and J. Rudolph , “Cdc25 Phosphatases and Cancer,” Chemistry & Biology 11 (2004): 1043–1051. 15324805 10.1016/j.chembiol.2004.07.007

[7] B. Aressy and B. Ducommun , “Cell Cycle Control by the CDC25 Phosphatases,” Anti‐Cancer Agents in Medicinal Chemistry 8 (2008): 818–824. 19075563 10.2174/187152008786847756

[8] P. Russell and P. Nurse , “cdc25+ Functions as an Inducer in the Mitotic Control of Fission Yeast,” Cell 45 (1986): 145–153. 3955656 10.1016/0092-8674(86)90546-5

[9] U. Strausfeld , J. C. Labbé , D. Fesquet , et al., “Dephosphorylation and Activation of a p34cdc2/Cyclin B Complex In Vitro by Human CDC25 Protein,” Nature 351 (1991): 242–245. 1828290 10.1038/351242a0

[10] C. Karlsson‐Rosenthal and J. B. A. Millar , “Cdc25: Mechanisms of Checkpoint Inhibition and Recovery,” Trends in Cell Biology 16 (2006): 285–292. 16682204 10.1016/j.tcb.2006.04.002

[11] A. Nagata , M. Igarashi , S. Jinno , K. Suto , and H. Okayama , “An Additional Homolog of the Fission Yeast cdc25+ Gene Occurs in Humans and Is Highly Expressed in Some Cancer Cells,” The New Biologist 3 (1991): 959–968. 1662986

[12] A. Capasso , C. Cerchia , C. Di Giovanni , et al., “Ligand‐Based Chemoinformatic Discovery of a Novel Small Molecule Inhibitor Targeting CDC25 Dual Specificity Phosphatases and Displaying In Vitro Efficacy against Melanoma Cells,” Oncotarget 6 (2015): 40202–40222. 26474275 10.18632/oncotarget.5473 PMC4741889

[13] I. Dakilah , A. Harb , E. Abu‐Gharbieh , et al., “Potential of CDC25 Phosphatases in Cancer Research and Treatment: Key to Precision Medicine,” Frontiers in Pharmacology 15 (2024): 1324001. 38313315 10.3389/fphar.2024.1324001 PMC10834672

[14] R. Boutros , C. Dozier , and B. Ducommun , “The when and Wheres of CDC25 Phosphatases,” Current Opinion in Cell Biology 18 (2006): 185–191. 16488126 10.1016/j.ceb.2006.02.003

[15] R. Boutros , V. Lobjois , and B. Ducommun , “CDC25 Phosphatases in Cancer Cells: Key Players? Good Targets?,” Nature Reviews Cancer 7 (2007): 495–507. 17568790 10.1038/nrc2169

[16] C. Lammer , S. Wagerer , R. Saffrich , D. Mertens , W. Ansorge , and I. Hoffmann , “The cdc25B Phosphatase Is Essential for the G2/M Phase Transition in Human Cells,” Journal of Cell Science 111 (1998): 2445–2453. 9683638 10.1242/jcs.111.16.2445

[17] S. Sur and D. K. Agrawal , “Phosphatases and Kinases Regulating CDC25 Activity in the Cell Cycle: Clinical Implications of CDC25 Overexpression and Potential Treatment Strategies,” Molecular and Cellular Biochemistry 416 (2016): 33–46. 27038604 10.1007/s11010-016-2693-2 PMC4862931

[18] J. Bonnet , P. Coopman , and M. C. Morris , “Characterization of Centrosomal Localization and Dynamics of Cdc25C Phosphatase in Mitosis,” Cell Cycle Georgetown Tex 7 (2008): 1991–1998. 18604163 10.4161/cc.7.13.6095

[19] A. K. Brenner , H. Reikvam , A. Lavecchia , and Ø. Bruserud , “Therapeutic Targeting the Cell Division Cycle 25 (CDC25) Phosphatases in Human Acute Myeloid Leukemia‐‐the Possibility to Target Several Kinases through Inhibition of the Various CDC25 Isoforms,” Molecules 19 (2014): 18414–18447. 25397735 10.3390/molecules191118414 PMC6270710

[20] E. B. Fauman , J. P. Cogswell , B. Lovejoy , et al., “Crystal Structure of the Catalytic Domain of the Human Cell Cycle Control Phosphatase, Cdc25A,” Cell 93 (1998): 617–625. 9604936 10.1016/s0092-8674(00)81190-3

[21] R. A. Reynolds , A. W. Yem , C. L. Wolfe , M. R. Deibel , C. G. Chidester , and K. D. Watenpaugh , “Crystal Structure of the Catalytic Subunit of Cdc25B Required for G2/M Phase Transition of the Cell Cycle,” Journal of Molecular Biology 293 (1999): 559–568. 10543950 10.1006/jmbi.1999.3168

[22] K. Liu , M. Zheng , R. Lu , et al., “The Role of CDC25C in Cell Cycle Regulation and Clinical Cancer Therapy: A Systematic Review,” Cancer Cell International 20 (2020): 213. 32518522 10.1186/s12935-020-01304-w PMC7268735

[23] R. J. Rowland , S. Korolchuk , M. Salamina , et al., “Cryo‐EM Structure of the CDK2‐Cyclin A‐CDC25A Complex,” Nature Communications 15 (2024): 6807. 10.1038/s41467-024-51135-w PMC11316097 39122719

[24] M.‐C. Brezak , M. Quaranta , O. Mondésert , et al., “A Novel Synthetic Inhibitor of CDC25 Phosphatases: BN82002,” Cancer Research 64 (2004): 3320–3325. 15126376 10.1158/0008-5472.can-03-3984

[25] M.‐C. Brezak , M. Quaranta , M.‐O. Contour‐Galcera , et al., “Inhibition of Human Tumor Cell Growth In Vivo by an Orally Bioavailable Inhibitor of CDC25 Phosphatases,” Molecular Cancer Therapeutics 4 (2005): 1378–1387. 16170030 10.1158/1535-7163.MCT-05-0168

[26] M. Brisson , C. Foster , P. Wipf , et al., “Independent Mechanistic Inhibition of cdc25 Phosphatases by a Natural Product Caulibugulone,” Molecular Pharmacology 71 (2007): 184–192. 17018577 10.1124/mol.106.028589

[27] X. Feng , L. Wang , Y. Zhou , et al., “Discovery and Characterization of a Novel Inhibitor of CDC25B, LGH00045,” Acta Pharmacologica Sinica 29 (2008): 1268–1274. 18817634 10.1111/j.1745-7254.2008.00841.x

[28] M.‐C. Brezak , A. Valette , M. Quaranta , et al., “IRC‐083864, a Novel Bis Quinone Inhibitor of CDC25 Phosphatases Active against Human Cancer Cells,” International Journal of Cancer 124 (2009): 1449–1456. 19065668 10.1002/ijc.24080

[29] A. Lavecchia , C. Di Giovanni , A. Pesapane , et al., “Discovery of New Inhibitors of Cdc25B Dual Specificity Phosphatases by Structure‐Based Virtual Screening,” Journal of Medicinal Chemistry 55 (2012): 4142–4158. 22524450 10.1021/jm201624h

[30] S. Zhang , Q. Jia , Q. Gao , X. Fan , Y. Weng , and Z. Su , “Dual‐Specificity Phosphatase CDC25B Was Inhibited by Natural Product HB‐21 Through Covalently Binding to the Active Site,” Frontiers in Chemistry 6 (2018): 531. 30555816 10.3389/fchem.2018.00531 PMC6282036

[31] C. Cerchia , R. Nasso , M. Mori , et al., “Discovery of Novel Naphthylphenylketone and Naphthylphenylamine Derivatives as Cell Division Cycle 25B (CDC25B) Phosphatase Inhibitors: Design, Synthesis, Inhibition Mechanism, and In Vitro Efficacy against Melanoma Cell Lines,” Journal of Medicinal Chemistry 62 (2019): 7089–7110. 31294975 10.1021/acs.jmedchem.9b00632

[32] Y. Nagaoka , P. Parvatkar , G. Hirai , and J. Ohkanda , “Design, Synthesis, and Functional Evaluation of Triazine‐Based Bivalent Agents that Simultaneously Target the Active Site and Hot Spot of Phosphatase Cdc25B,” Bioorganic & Medicinal Chemistry Letters 48 (2021): 128265. 34273487 10.1016/j.bmcl.2021.128265

[33] N. Liu , Y. Tao , P. Zhan , X. Liu , and Y. Song , “Discovery of New Inhibitors of Cdc25B Phosphatases by Molecular Docking‐Based Virtual Screening,” Journal of Molecular Structure 1299 (2024): 137161.

[34] G. Lund , S. Dudkin , D. Borkin , W. Ni , J. Grembecka , and T. Cierpicki , “Inhibition of CDC25B Phosphatase through Disruption of Protein‐Protein Interaction,” ACS Chemical Biology 10 (2015): 390–394. 25423142 10.1021/cb500883h PMC4340349

[35] F. Aliotta , R. Nasso , R. Rullo , et al., “Inhibition Mechanism of Naphthylphenylamine Derivatives Acting on the CDC25B Dual Phosphatase and Analysis of the Molecular Processes Involved in the High Cytotoxicity Exerted by One Selected Derivative in Melanoma Cells,” Journal of Enzyme Inhibition and Medicinal Chemistry 35 (2020): 1866–1878. 32990107 10.1080/14756366.2020.1819257 PMC7580834

[36] E. B. Gottlin , X. Xu , D. M. Epstein , et al., “Kinetic Analysis of the Catalytic Domain of Human Cdc25B,” The Journal of Biological Chemistry 271 (1996): 27445–27449. 8910325 10.1074/jbc.271.44.27445

[37] P. Turowski , C. Franckhauser , M. C. Morris , P. Vaglio , A. Fernandez , and N. J. C. Lamb , “Functional cdc25C Dual‐Specificity Phosphatase Is Required for S‐Phase Entry in Human Cells,” Molecular Biology of the Cell 14 (2003): 2984–2998. 12857880 10.1091/mbc.E02-08-0515 PMC165692

[38] R. J. Tomko and J. S. Lazo , “Multimodal Control of Cdc25A by Nitrosative Stress,” Cancer Research 68 (2008): 7457–7465. 18794133 10.1158/0008-5472.CAN-08-0625 PMC2680243

[39] P. A. Johnston , C. A. Foster , M. B. Tierno , et al., “Cdc25B Dual‐Specificity Phosphatase Inhibitors Identified in a High‐Throughput Screen of the NIH Compound Library,” Assay and Drug Development Technologies 7 (2009): 250–265. 19530895 10.1089/adt.2008.186 PMC2956648

[40] A. J. McCoy , R. W. Grosse‐Kunstleve , P. D. Adams , M. D. Winn , L. C. Storoni , and R. J. Read , “Phaser Crystallographic Software,” Journal of Applied Crystallography 40 (2007): 658–674. 19461840 10.1107/S0021889807021206 PMC2483472

[41] J. Sohn , J. M. Parks , G. Buhrman , et al., “Experimental Validation of the Docking Orientation of Cdc25 with Its Cdk2‐CycA Protein Substrate,” Biochemistry 44 (2005): 16563–16573. 16342947 10.1021/bi0516879

[42] G. Langer , S. X. Cohen , V. S. Lamzin , and A. Perrakis , “Automated Macromolecular Model Building for X‐ray Crystallography Using ARP/wARP Version 7,” Nature Protocols 3 (2008): 1171–1179. 18600222 10.1038/nprot.2008.91 PMC2582149

[43] G. N. Murshudov , P. Skubák , A. A. Lebedev , et al., “REFMAC5 for the Refinement of Macromolecular Crystal Structures,” Acta Crystallographica Section D Biological Crystallography 67 (2011): 355–367. 21460454 10.1107/S0907444911001314 PMC3069751

[44] J. B. Grimm , L. M. Heckman , and L. D. Lavis , Progress in Molecular Biology and Translational Science, ed. M. C. Morris , (Academic Press, 2013), 1–34. 10.1016/B978-0-12-386932-6.00001-6 23244787

[45] F. Yan , K. Fan , Z. Bai , et al., “Fluorescein Applications as Fluorescent Probes for the Detection of Analytes,” TrAC Trends in Analytical Chemistry 97 (2017): 15–35.

[46] M. Rajasekar , “Recent Development in Fluorescein Derivatives,” Journal of Molecular Structure 1224 (2021): 129085.

[47] L. Tautz and E. A. Sergienko , “High‐Throughput Screening for Protein Tyrosine Phosphatase Activity Modulators,” Methods in Molecular Biology 1053 (2013): 223–240. 23860657 10.1007/978-1-62703-562-0_14 PMC8148610

[48] J. Rudolph , “Targeting the Neighbor's Pool,” Molecular Pharmacology 66 (2004): 780–782. 15258253 10.1124/mol.104.004788

[49] A. Lavecchia , A. Coluccia , C. Di Giovanni , and E. Novellino , “Cdc25B Phosphatase Inhibitors in Cancer Therapy: Latest Developments, Trends and Medicinal Chemistry Perspective,” Anti‐Cancer Agents in Medicinal Chemistry 8 (2008): 843–856. 19075567 10.2174/187152008786847783

[50] J. S. Lazo , D. C. Aslan , E. C. Southwick , et al., “Discovery and Biological Evaluation of a New Family of Potent Inhibitors of the Dual Specificity Protein Phosphatase Cdc25,” Journal of Medicinal Chemistry 44 (2001): 4042–4049. 11708908 10.1021/jm0102046

[51] Y. Ge , M. van der Kamp , M. Malaisree , D. Liu , Y. Liu , and A. J. Mulholland , “Identification of the Quinolinedione Inhibitor Binding Site in Cdc25 Phosphatase B through Docking and Molecular Dynamics Simulations,” Journal of Computer‐Aided Molecular Design 31 (2017): 995–1007. 28994029 10.1007/s10822-017-0073-y

[52] R. S. R. Sayegh , F. K. Tamaki , S. R. Marana , R. K. Salinas , and G. M. Arantes , “Conformational Flexibility of the Complete Catalytic Domain of Cdc25B Phosphatases,” Proteins 84 (2016): 1567–1575. 27410025 10.1002/prot.25100

[53] J. Lin , D. C. Sahakian , S. M. F. de Morais , J. J. Xu , R. J. Polzer , and S. M. Winter , “The Role of Absorption, Distribution, Metabolism, Excretion and Toxicity in Drug Discovery,” Current Topics in Medicinal Chemistry 3 (2003): 1125–1154. 12769713 10.2174/1568026033452096

[54] P. Evans , “Scaling and Assessment of Data Quality,” Acta Crystallographica Section D Biological Crystallography 62 (2006): 72–82. 16369096 10.1107/S0907444905036693

[55] W. Kabsch , “XDS,” Acta Crystallographica Section D Biological Crystallography 66 (2010): 125–132. 20124692 10.1107/S0907444909047337 PMC2815665

[56] M. D. Winn , C. C. Ballard , K. D. Cowtan , et al., “Overview of the CCP4 Suite and Current Developments,” Acta Crystallographica Section D Biological Crystallography 67 (2011): 235–242. 21460441 10.1107/S0907444910045749 PMC3069738

[57] C. Vonrhein , C. Flensburg , P. Keller , et al., “Data Processing and Analysis with the autoPROC Toolbox,” Acta Crystallographica Section D Biological Crystallography 67 (2011): 293–302. 21460447 10.1107/S0907444911007773 PMC3069744

[58] P. R. Evans and G. N. Murshudov , “How Good Are My Data and What Is the Resolution?,” Acta Crystallographica Section D Biological Crystallography 69 (2013): 1204–1214. 23793146 10.1107/S0907444913000061 PMC3689523

[59] P. Emsley , B. Lohkamp , W. G. Scott , and K. Cowtan , “Features and Development of Coot,” Acta Crystallographica Section D Biological Crystallography 66 (2010): 486–501. 20383002 10.1107/S0907444910007493 PMC2852313

[60] E. Krissinel and K. Henrick , “Inference of Macromolecular Assemblies from Crystalline State,” Journal of Molecular Biology 372 (2007): 774–797. 17681537 10.1016/j.jmb.2007.05.022

[61] R. A. Laskowski and M. B. Swindells , “LigPlot+: Multiple Ligand‐Protein Interaction Diagrams for Drug Discovery,” Journal of Chemical Information and Modeling 51 (2011): 2778–2786. 21919503 10.1021/ci200227u

