# Supplementary material for: The Binding of 3‐O‐Methylfluorescein Phosphate to the Catalytic Domain of the Human CDC25B Phosphatase: A Structural Investigation
Source: Chembiochem. 2026 Mar 29;27(7):e202600010. doi: 10.1002/cbic.202600010 (PMC13033960; doi:10.1002/cbic.202600010)
Supplement: Supplementary file 1 — Supplementary Material [file CBIC-27-e202600010-s001.pdf]

# Supporting information for:

# The Binding of 3-O-Methylfluorescein Phosphate to the Catalytic Domain of the Human CDC25B Phosphatase: A Structural Investigation

Romualdo Troisi,*[a] Rosario Rullo,[b] Valeria Napolitano,[c,d] Grzegorz M. Popowicz,[c,d] Emmanuele De Vendittis,[e] and Filomena Sica*[a]

[a] Department of Chemical Sciences, University of Naples Federico II, Complesso Universitario di Monte Sant'Angelo, via Cintia, 80126 Naples, Italy;
[b] Institute for the Animal Production Systems in the Mediterranean Environment, Consiglio Nazionale delle Ricerche, Piazzale E. Fermi 1, 80055 Portici, Italy;
[c] Institute of Structural Biology, Helmholtz Zentrum München, Ingolstädter Landstraße 1, 85764 Neuherberg, Germany;
[d] Biomolecular NMR and Center for Integrated Protein Science Munich at Department Chemie, Technical University of Munich, Lichtenbergstraße 4, 85747 Garching, Germany;
[e] Department of Molecular Medicine and Medical Biotechnologies, University of Naples Federico II, via S. Pansini 5, 80131 Naples, Italy.

* Corresponding authors: R. Troisi, romualdo.troisi@unina.it; F. Sica, filomena.sica@unina.it

## *Supplemental Tables*

**Table S1.** Melting temperature of CDC25B and its mutant CDC25B-S in different conditions.

| Protein | Condition | Ligand (3-OMFP) | $T_m$ (°C) |
|---|---|---|---|
| CDC25B | 40 mM Tris-HCl pH 8.0,<br>40 mM NaCl, 1.7 mM dithiothreitol (DTT) | - | 46 ± 1 |
| CDC25B-S | 40 mM Tris-HCl pH 8.0,<br>40 mM NaCl, 1.7 mM DTT | - | 47 ± 1 |
| CDC25B-S | 40 mM Tris-HCl pH 8.0,<br>40 mM NaCl, 1.7 mM DTT,<br>2.3% v/v $CH_3OH$ | - | 48 ± 1 |
| CDC25B-S | 40 mM Tris-HCl pH 8.0,<br>40 mM NaCl, 1.7 mM DTT,<br>2.3% v/v $CH_3OH$ | 5-fold molar excess | 52 ± 1 |

**Table S2.** Data collection and refinement statistics. Values in brackets refer to the highest resolution shell.

| | 3-OMFP/CDC25B-S | CDC25B-S |
|---|---|---|
| ***Crystal data*** | | |
| **Crystallization condition** | 3.8 M NaCl<br>0.1 M HEPES pH 7.5<br>1.2% v/v 2-propanol | 2.0 M ammonium sulphate<br>0.5 M NaCl<br>0.1 M Tris-HCl pH 7.5<br>0.5 mM TCEP |
| **Space group** | $P2_12_12_1$ | $P2_12_12_1$ |
| **Unit-cell parameters** | | |
| **a, b, c (Å)** | 56.54, 66.32, 68.40 | 52.13, 71.20, 72.90 |
| **α, β, γ (deg)** | 90.00, 90.00, 90.00 | 90.00, 90.00, 90.00 |
| **No. of molecules in the asymmetric unit** | 1 | 1 |
| ***Data collection*** | | |
| **Resolution limits (Å)** | 47.61 – 2.04 (2.10 – 2.04) | 42.40 – 1.34 (1.36 – 1.34) |
| **No. of observations** | 190158 (6479) | 271536 (14904) |
| **No. of unique reflections** | 16632 (1117) | 57489 (3023) |
| **Completeness (%)** | 98.6 (87.5) | 93.6 (100.0) |
| **$\langle I/\sigma(I) \rangle$** | 15.3 (1.4) | 13.6 (2.2) |
| **Average multiplicity** | 11.4 (5.8) | 4.7 (4.9) |
| **$CC_{1/2}$** | 1.0 (0.5) | 1.0 (0.7) |
| ***Refinement*** | | |
| **Resolution limits (Å)** | 43.61 – 2.04 | 42.40 – 1.34 |
| **No. of reflections** | 15694 | 54633 |
| **$R_{factor}/R_{free}$** | 0.195/0.214 | 0.157/0.175 |
| **No. of atoms** | 1705 | 1939 |
| **Mean B value (Å$^2$)** | 36.9 | 20.8 |
| **RMSD from ideal values** | | |
| **Bond lengths (Å)** | 0.004 | 0.015 |
| **Bond angles (°)** | 1.430 | 2.261 |
| **Ramachandran plot, residues in (%)** | | |
| **Most favored region** | 94.1 | 96.5 |
| **Additionally allowed region** | 5.9 | 3.5 |
| **Generously allowed region** | 0 | 0 |
| ***PDB code*** | 9T0A | 9T09 |

**Table S3.** Interface interactions and buried area in 3-OMFP/CDC25B-S complex. Atoms involved in hydrogen bond interactions are indicated as superscripts.

| | **R-enantiomer** | **S-enantiomer** |
|---|---|---|
| **Buried area (Å$^2$)** | 291 | 285 |
| **Hydrogen bonds** | 3-OMFP$^{O4}$-Glu474$^{N}$<br>3-OMFP$^{O4}$-Arg479$^{N\varepsilon}$<br>3-OMFP$^{O4}$-Arg479$^{N\eta 2}$<br>3-OMFP$^{O5}$-Ser473$^{O\gamma}$<br>3-OMFP$^{O5}$-Glu478$^{N}$<br>3-OMFP$^{O5}$-Arg479$^{N}$<br>3-OMFP$^{O6}$-Phe475$^{N}$<br>3-OMFP$^{O6}$-Ser476$^{N}$<br>3-OMFP$^{O6}$-Ser477$^{N}$<br><br><br>3-OMFP$^{O8}$-Arg544$^{N\varepsilon}$ | 3-OMFP$^{O4}$-Glu474$^{N}$<br>3-OMFP$^{O4}$-Arg479$^{N\varepsilon}$<br>3-OMFP$^{O4}$-Arg479$^{N\eta 2}$<br>3-OMFP$^{O5}$-Ser473$^{O\gamma}$<br>3-OMFP$^{O5}$-Glu478$^{N}$<br>3-OMFP$^{O5}$-Arg479$^{N}$<br>3-OMFP$^{O6}$-Phe475$^{N}$<br>3-OMFP$^{O6}$-Ser476$^{N}$<br>3-OMFP$^{O6}$-Ser477$^{N}$<br>3-OMFP$^{O7}$-Arg482$^{N\eta 1}$<br>3-OMFP$^{O8}$-Tyr428$^{O\eta}$ |
| **Protein residues involved in hydrophobic interactions** | Tyr428<br>Glu478<br>Arg479<br>Arg482<br>Met531<br><br>Thr547 | Tyr428<br>Glu478<br>Arg479<br><br>Met531<br>Leu540<br><br>Arg544 |

***Supplemental Figures***

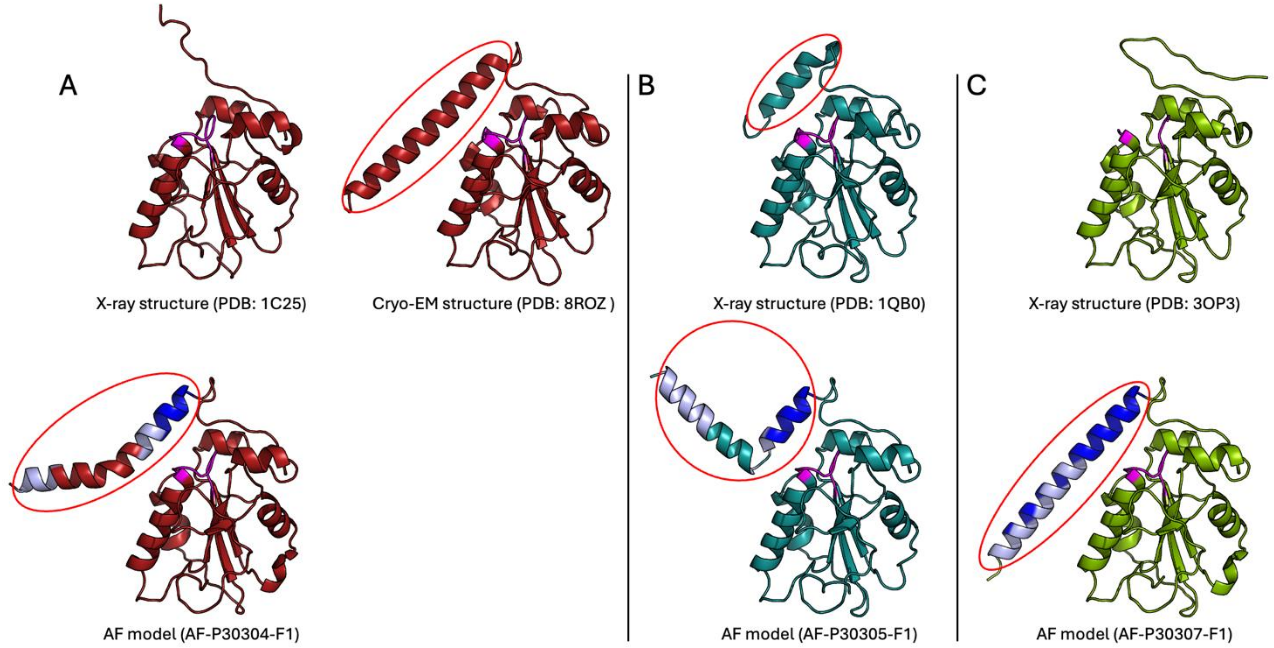


**Figure S1.** Experimental structures and predicted models of the catalytic domains of A) CDC25A, B) CDC25B, and C) CDC25C. The $HCX_5R$ motifs of the active sites are in magenta, while the C-terminal helices are circled. In the AlphaFold (AF) models, the C-terminal helix regions predicted with high and very high confidence are in light and dark blue, respectively.

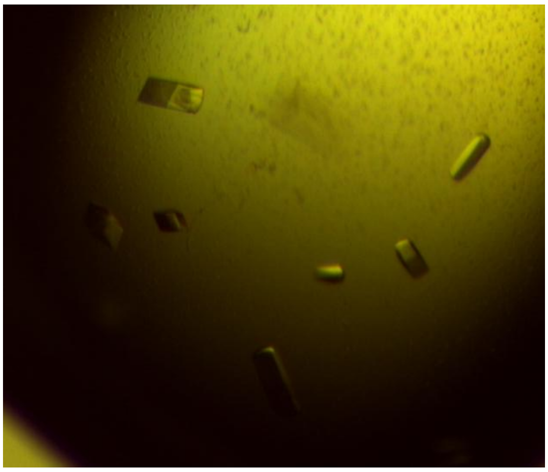

**Figure S2.** Crystals of the 3-OMFP/CDC25B-S complex, grown in 3.8 M NaCl, 0.1 M HEPES pH 7.5, and 1.2% v/v 2-propanol.

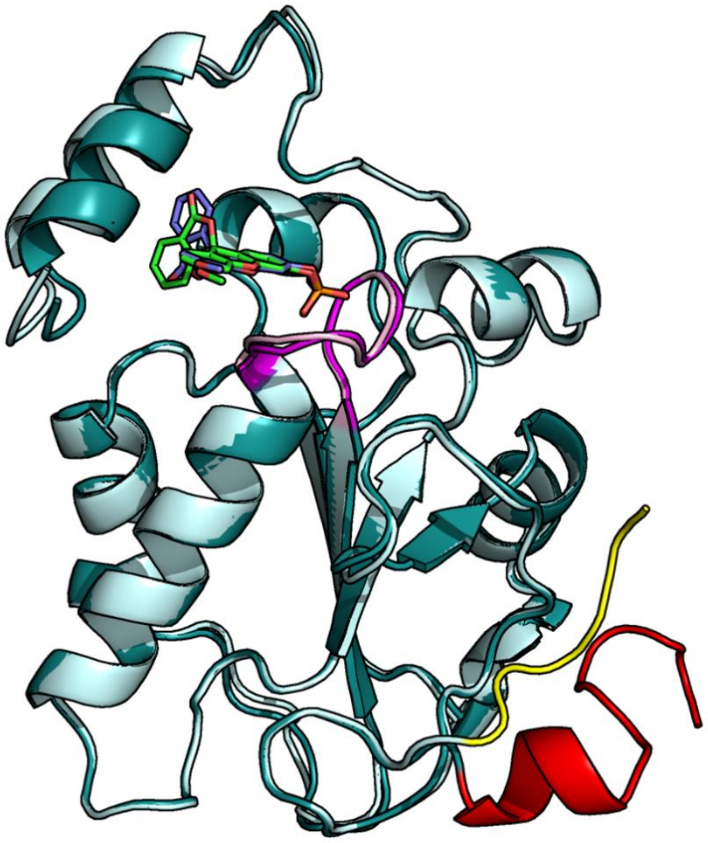

**Figure S3.** Superimposition of the structures reported here for the 3-OMFP/CDC25B-S complex (deep teal) and the free CDC25B-S protein (light cyan), with the N-terminal expression tags highlighted in red and yellow and the $HCX_5R$ motifs in magenta and light pink, respectively. The R- and S-enantiomers of 3-OMFP are green and violet, respectively.

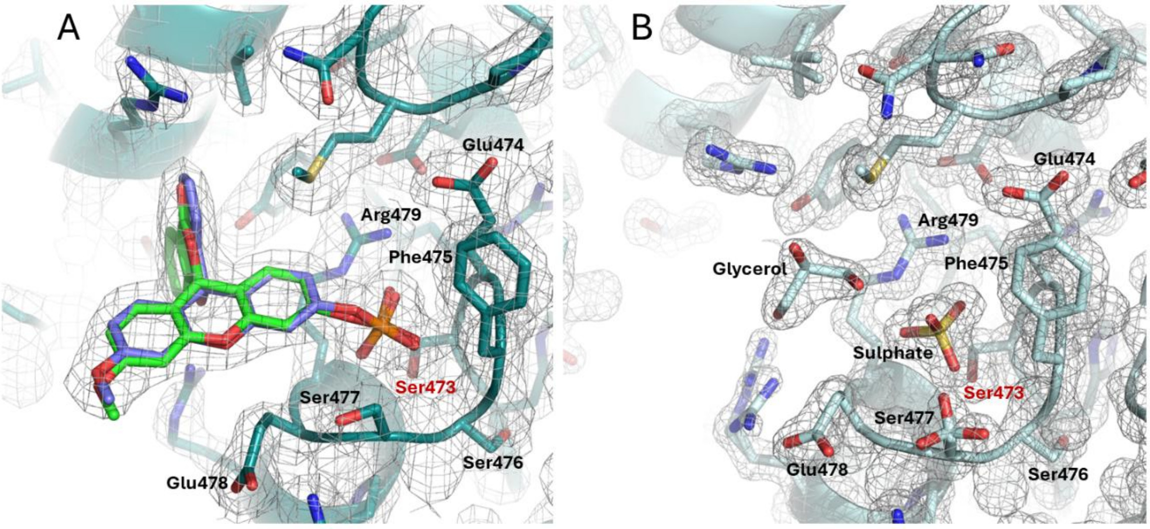


**Figure S4.** Details of the active site in the structures of A) the 3-OMFP/CDC25B-S complex and B) the ligand-free CDC25B-S protein. In this latter, a sulphate ion, originating from crystallization conditions, binds the active site. A glycerol molecule, deriving from the cryoprotection procedure, is also observed. $2F_o$-$F_c$ electron density maps (grey) are contoured at 1.0 σ level. The Ser473 residue, which replaces the catalytic cysteine, is labelled in red. In A), the R- and S-enantiomers of 3-OMFP are shown in green and violet, respectively.

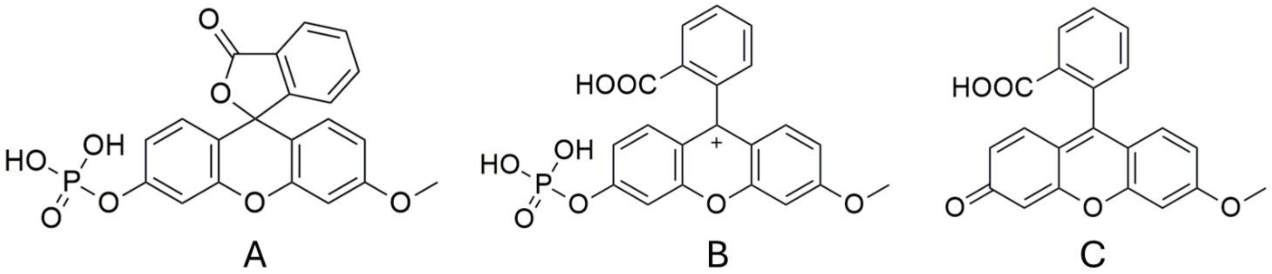


**Figure S5.** Chemical structures of 3-OMFP in the A) “closed” lactone form and B) “open” cationic form. C) Chemical structure of the “open” quinoid form of the highly fluorescent 3-O-methylfluorescein (3-OMF).

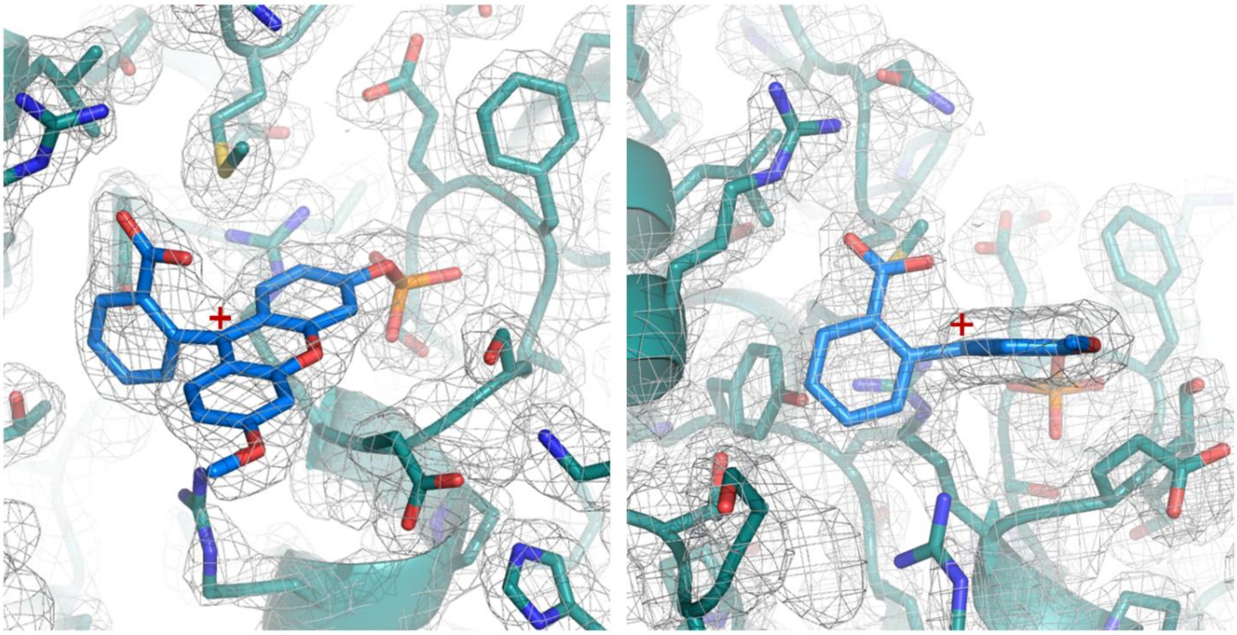

**Figure S6.** Detail of two orientations of the 3-OMFP/CDC25B-S structure in which the 3-OMFP molecule has been modeled in an “open” cationic form. The $2F_o$-$F_c$ electron density map (grey) is contoured at 1.0 σ level. In contrast to what would be typically expected for an “open” cationic form, the carboxylic group appears nearly coplanar with the lateral aromatic ring to which it is attached, and the cationic carbon (indicated with a red plus) does not adopt a strictly trigonal planar geometry.

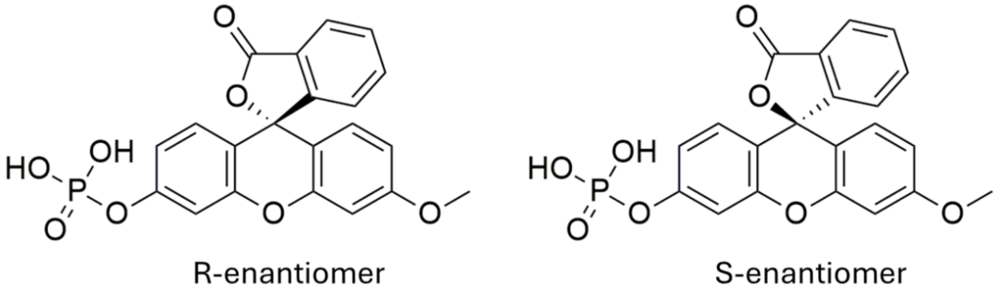


**Figure S7.** Chemical structures of the two enantiomers of 3-OMFP lactone form.

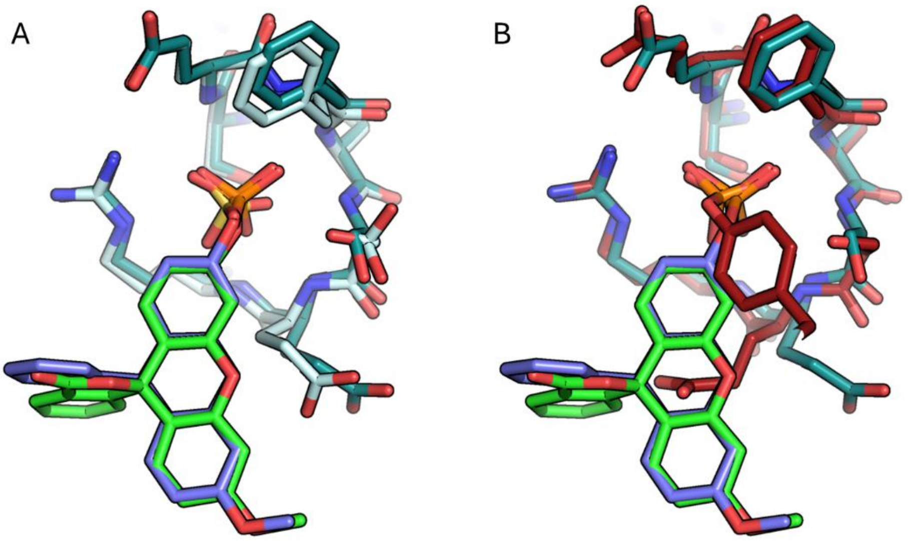


**Figure S8.** Comparison of the binding mode of the phosphate group of 3-OMFP in 3-OMFP/CDC25B-S structure (deep teal) with A) the sulfate ion observed in the ligand-free CDC25B-S crystal structure (light cyan) and B) the phosphorylated tyrosine of CDK2 in the cryo-EM structure of the CDK2-cyclin A-CDC25A complex (dark red, PDB code: 8ROZ). The R- and S-enantiomers of 3-OMFP are shown in green and violet, respectively.

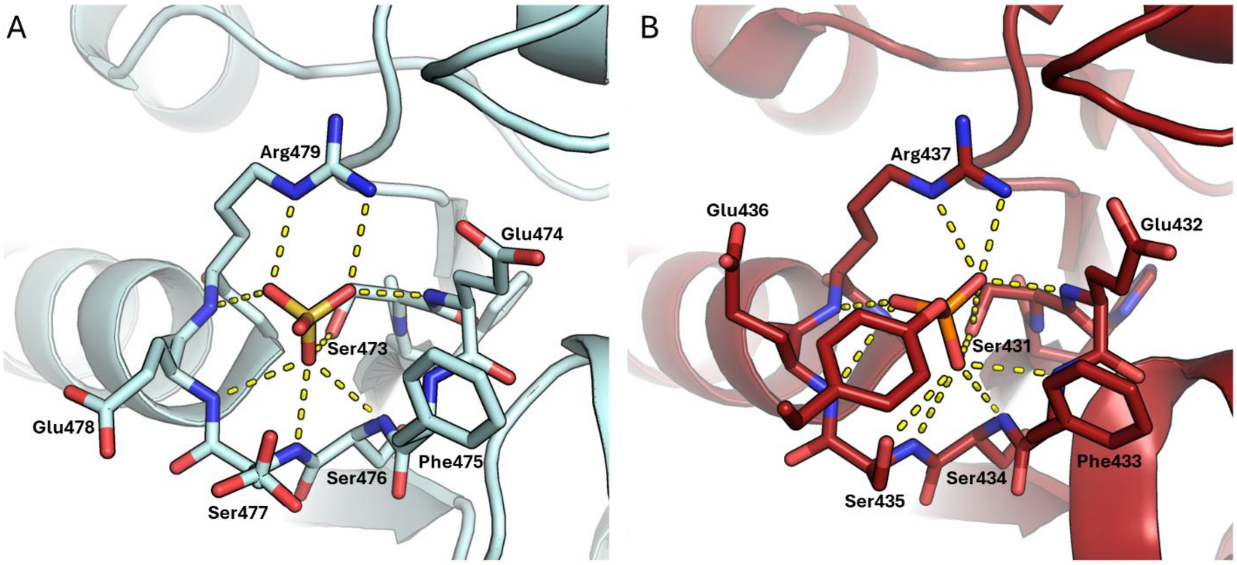


**Figure S9.** Contacts between A) the CDC25B-S active site residues and the sulfate ion observed in the ligand-free CDC25B-S crystal structure and B) the CDC25A active site residues and the CDK2 phosphorylated tyrosine in the cryo-EM structure of the CDK2-cyclin A-CDC25A complex (PDB code: 8ROZ).

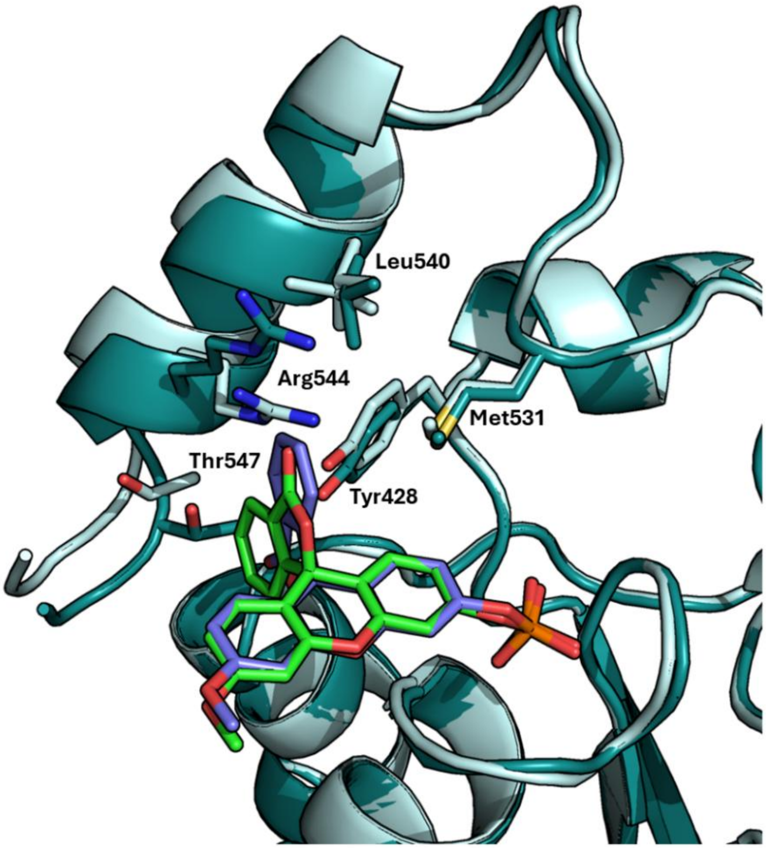


**Figure S10.** Superimposition of the 3-OMFP/CDC25B-S complex (deep teal) and the free CDC25B-S protein (light cyan), highlighting the conformational rearrangement of Arg544 side chain to accommodate the bicyclic lactone group of 3-OMFP. The R- and S-enantiomers of 3-OMFP are shown in green and violet, respectively.

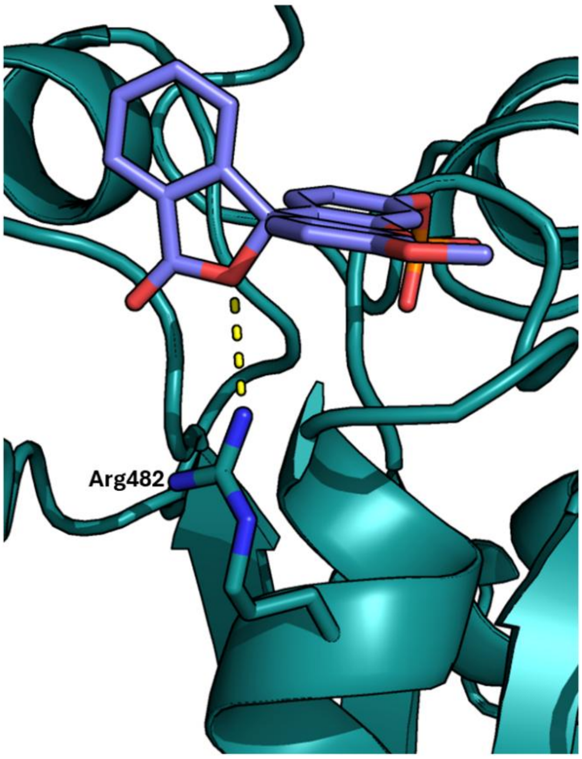


**Figure S11.** Transient interaction between the S-enantiomer of 3-OMFP and Arg482 of CDC25B-S.

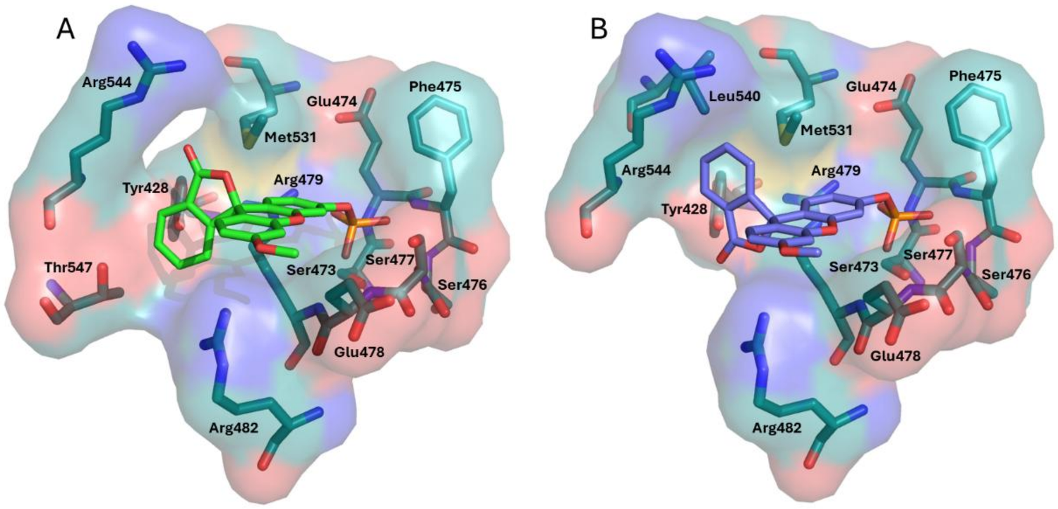


**Figure S12.** The shallow surface active-site pocket of CDC25B-S: details of the binding of the A) R-enantiomer and B) S-enantiomer of 3-OMFP, highlighting the high solvent exposure of the ligand and the predominantly polar nature of the interacting protein residues.

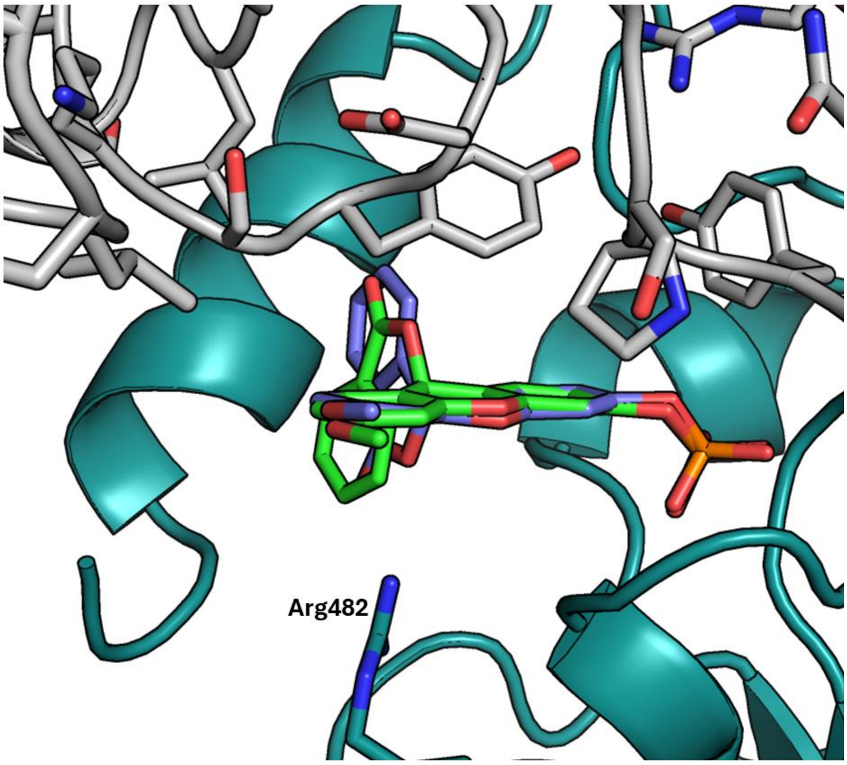


**Figure S13.** Crystal packing contacts involving the 3-OMFP molecule. The symmetry-related CDC25B-S molecule is shown in light gray. The R- and S-enantiomers of 3-OMFP are shown in green and violet, respectively.
